# Frequency and type of domestic injuries among children during COVID-19 lockdown: what changes from the past? An Italian multicentre cohort study

**DOI:** 10.1007/s00431-023-04990-6

**Published:** 2023-05-15

**Authors:** Daiana Bezzini, Marcello Lanari, Alessandro Amaddeo, Melodie O. Aricò, Emanuele Castagno, Gabriella Cherchi, Giulia Giacomini, Giulia Graziani, Salvatore Grosso, Ilaria Liguoro, Francesca Lombardi, Sergio Manieri, Laura Moschettini, Francesca Parisi, Antonino Reale, Giulia Romanisio, Davide Silvagni, Irene Schiavetti, “Keep Me Safe” study group, Alberto Gaiero, Alessandra Iacono, Alessandro Amaddeo, Alessandro Canetto, Alice Fachin, Angela Demarco, Annalisa Lo Sasso, Annalisa Rossetti, Antonino Reale, Arianna Dagri, Carmela G. Raffaele, Chiara Ghizzi, Claudia Bondone, Daiana Bezzini, Daniele Zama, Davide Silvagni, Elisa Pala, Elisabetta Pangallo, Emanuele Castagno, Enrico Valerio, Enrico Valletta, Federico Marchetti, Francesca Lombardi, Francesca Nicolardi, Francesca Parisi, Francesco Medici, Francesco Silenzi, Gabriella Cherchi, Giulia Ceccarini, Giulia Giacomini, Giulia Graziani, Giulia Romanisio, Ilaria Corsini, Ilaria Liguoro, Irene Frigo, Irene Raffaldi, Irene Schiavetti, Laura Andreozzi, Laura Moschettini, Laura Penta, Luca Bianchini, Luciana Romaniello, Manuel Murciano, Manuela Pagano, Marcello Lanari, Maria Chiara Supino, Maria Pia Mirauda, Martina Scilipoti, Matteo Calvi, Melodie O. Aricò, Monia Gennari, Nicoletta Della Vecchia, Paolo Biban, Paolo Tarlazzi, Raffaele Pecoraro, Raffaella Nacca, Rosa Francavilla, Rosa Lapolla, Salvatore Grosso, Sergio Manieri, Silvia Carlassara, Simone Ajello, Stefano Masi, Viola Carzaniga

**Affiliations:** 1 Department of Life Sciences, University of Siena, via Aldo Moro 2, 53100 Siena, Italy; 2 Pediatric Emergency Unit, IRCCS Azienda Ospedaliera Universitaria di Bologna, Bologna, Italy; 3 Institute for Maternal and Child Health, IRCCS “Burlo Garofolo”, Trieste, Italy; 4 Pediatric Department, G.B. Morgagni - L. Pierantoni Hospital, Forlì, Italy; 5 Department of Paediatric Emergency, Regina Margherita Children’s Hospital - A.O.U. Città della Salute e della Scienza di Torino, Turin, Italy; 6 Ospedale San Michele, ARNAS “G.Brotzu”, Cagliari, Italy; 7 IRCCS Meyer University Children’s Hospital, Florence, Italy; 8 Department of Paediatrics, Santa Maria delle Croci Hospital, Ravenna, Italy; 9 Clinical Paediatrics, Department of Molecular Medicine and Development, University of Siena, Siena, Italy; 10 Division of Paediatrics, Department of Medicine (DAME), University of Udine, Udine, Italy; 11 Pediatric Department, Maggiore Hospital Carlo Alberto Pizzardi, Bologna AUSL, Bologna, Italy; 12 Department of Paediatrics, San Carlo Hospital, Potenza, Italy; 13 Pediatric Department, University of Milano Bicocca, Milan, Italy; 14 Department of Medicine and Surgery, Pediatric Clinic, University of Perugia, Perugia, Italy; 15 Department of Emergency and General Paediatrics, Bambino Gesù Children’s Hospital, IRCCS, Rome, Italy; 16 Pediatric and Neonatology Unit, ASL 2 Ospedale San Paolo, Savona, Italy; 17 Department of Paediatric Emergency, Azienda Ospedaliera Universitaria Integrata Verona, Verona, Italy; 18 Department of Health Sciences, University of Genoa, Genoa, Italy; 19 Pediatric Clinic, “Santa Maria della Misericordia” University Hospital - Azienda Sanitaria Universitaria Friuli Centrale, Udine, Italy

**Keywords:** Home, Accident, Incidence, Hospitalization

## Abstract

Accidents are the main cause of injury in children, more than half events happen at home. Aims of this study were to assess if SARS-CoV-2 lockdown influence emergency department (ED) visits due to children domestic accident (DAs) and to identify factors associated with hospitalization. This was a multicentre, observational, and retrospective cohort study involving 16 EDs in Italy and enrolling children (3–13 years) receiving a visit in ED during March–June 2019 and March–June 2020. Risk factors for hospitalization were identified by logistic regression models. In total, 8860 ED visits due to domestic accidents in children occurred before (4380) and during (4480) lockdown, with a mean incidence of DA of 5.6% in 2019 and 17.9% in 2020 (*p* < 0.001) (*IRR*: 3.16; *p* < 0.001). The risk of hospitalization was influenced by the type of occurred accident, with fourfold higher for poisoning and twofold lower risk for stab-wound ones. In addition, a higher risk was reported for lockdown period vs 2019 (*OR*: 1.9; *p* < 0.001), males (*OR*: 1.4; *p* < 0.001), and it increased with age (*OR*: 1.1; *p* < 0.001).

*    Conclusions*: The main limitation of this study is the retrospective collection of data, available only for patients who presented at the hospital. This does highlight possible differences in the total number of incidents that truly occurred. In any case, the COVID-19 lockdown had a high impact on the frequency of DAs and on hospitalization. A public health campaign aimed at caregivers would be necessary to minimize possible risks at home.

**Table:** 

**What is Known:** • *In Italy, domestic accidents are the second leading cause of paediatric mortality after cancer.* *• During the first SARS-CoV-2 lockdown in 2020, a sharp decrease in the total number of Emergency Departments visits for all causes was observed, both in children and in adults.*
**What is New:** *• During the first SARS-CoV-2 lockdown in 2020, domestic accidents involving children increased threefold from the previous year.* *• Higher risk of hospitalization was showed in minors accessing during 2020 vs 2019, in males than in females and it increased with advancing age. Considering the type of injury, a significant higher risk of hospitalization for poisoning was observed.*

**What is Known:**

• *In Italy, domestic accidents are the second leading cause of paediatric mortality after cancer.*

*• During the first SARS-CoV-2 lockdown in 2020, a sharp decrease in the total number of Emergency Departments visits for all causes was observed, both in children and in adults.*

**What is New:**

*• During the first SARS-CoV-2 lockdown in 2020, domestic accidents involving children increased threefold from the previous year.*

*• Higher risk of hospitalization was showed in minors accessing during 2020 vs 2019, in males than in females and it increased with advancing age. Considering the type of injury, a significant higher risk of hospitalization for poisoning was observed.*

## Introduction

Accidents are the main cause of injury and even death or disability in children. It is estimated that ten million children are injured victims [1] and 950,000 dies from accidents each year [2]. Most studies on childhood accidents indicate that more of half events are related to domestic environment, mainly due to general negligence of home safety [1].

In Italy, domestic accidents (DAs) are the second leading cause of paediatric mortality after cancer, accounting for more than 20% of all deaths and representing 75% of the total accidents. Every year, 350,000 children under 14 years of age receive at least one visit in an emergency department (ED) due to this type of injuries, with a predominance of males over females [3]. Even so, only 8% of caregiver living with children are aware of the risk of home injury [4].

From 2020, the scenario was radically changed due to the SARS-CoV-2 pandemic. Several reports have shown that the number of ED visits has decreased worldwide, also in Italy, due to a reduction of infectious diseases and to the caregivers’ fear to risk exposure to SARS-CoV-2 in a health-care setting [5].

On the other hand, from March 2020, all schools and childcare services were closed, with people discouraged from leaving the home, radically changing time spent in it.

The main aim was to investigate if SARS-CoV-2 lockdown affects frequency, severity, and type of ED visits due to DAs occurred in children in a large-scale multicentric study. A secondary aim was to evaluate which factors resulted associated with hospitalization.

## Methods

This was a multicentre, observational, and retrospective cohort. The Ethics Committee of University of Siena has confirmed that no ethical approval is required.

### Study population

Children aged between 3 and 13 years were included since this age group was the most affected one by the lockdown in terms of the difference in staying at home compared to the previous year.

To obtain a realistic picture of the Italian Country, several paediatric EDs covering most regions were included in the study.

### Data collection

DA was defined as “any event occurring inside the house or in immediate surroundings of house that resulted in injury” [6].

The lockdown period from 1st March 2020 to 30th June 2020 was compared with the same period in 2019. Whenever possible, we assessed the total number of visits in ED, among children between 3 and 13 years of age, regardless of the cause.

Demographic data, injury type, and characteristics (including trauma, poisoning, stab wound, burns, and presence of foreign bodies) and patients’ management information were retrieved for all patients with a diagnosis of DA from the ED electronic database of each enrolled hospital. The intentional or abusive trauma were included, too.

Regarding priority code for accessing the ED visits, some hospitals use the new Italian system with 5 colours (including white, green, blue, orange, and red), but the others use the old system with 4 colours (white, green, yellow, red). For this reason, we decided to merge the blue and green codes of the new system into the green code of the old system, and we considered the orange code in the new system as the yellow in the old one. Here are the features of each colour: white, non-urgent problem; green, stable condition with no evolutional risk; yellow, risk of impairment of vital functions; red, interruption or impairment of one or more vital functions.

### Statistical analysis

Categorical variables were described as frequency with percentages, and continuous variables were described as mean with standard deviation and range (min–max).

Differences in continuous variables were assessed by Student’s *t* test or corresponding non-parametric Mann–Whitney *U* test based on data distribution. Any relationship between discrete categorical data was explored by the chi-square test, or Fisher’s exact test, as appropriate.

Univariate and subsequent multivariate logistic regression models (adjusted for age and sex of the patients) were fitted to search for risk factors associated with hospitalization. The multivariate model included as independent predictor variables only factors with a *p* value < 0.10 at univariate analysis. Statistical significance was set at 0.05.

## Results

Overall, 8860 visits for DA were recorded in 16 EDs, of which 4380 in 2019 and 4480 in 2020. Considering total ED visits, among children between 3 and 13 years of age, for all causes, 69,160 visits occurred in 2019 and 23,556 in 2020 (data available for 12 enrolled EDs), with a mean incidence of DA of 5.6% (CI 95%: 5.4–5.8) in 2019 and 17.9% (CI 95%: 17.3–18.4) in 2020 (*p* < 0.001) and an incidence rate ratio (IRR) of 3.16 (3.02–3.30; *p* < 0.01) (Appendix — Table 4).

**Table 1:** Characteristics of the sample and hospital access

		**2019**	**2020**	***OR* (95% CI); *p***
Sex — *n* (%)	Females	1919 (43.8%)	1984 (44.3%)	Males vs females 0.98 (0.90–1.07); 0.65
	Males	2461 (56.2%)	2496 (55.7%)	
Age, years — mean ± SD		7.1 ± 3.18	6.9 ± 3.02	0.98 (0.97–0.99); 0.001
Citizenship — *n* (%)	Italian	3940 (90.0%)	4143 (93.0%)	Italian vs foreign: 1.47 (1.26–1.71); < 0.001
	Foreign	439 (10.0%)	314 (7.0%)	
Time of access — *n* (%)	12 a.m.–6 a.m	14 (0.3%)	6 (0.1%)	Day vs night (*from 6 a.m. to 6 p.m. vs from 6 p.m. to 6 a.m.*) 1.21 (1.11–1.32); < 0.001
	6 a.m.–12 p.m	811 (19.4%)	727 (17.0%)	
	12 p.m.–6 p.m	1342 (32.1%)	1683 (39.3%)	
	6 p.m.–12 a.m	2012 (48.1%)	1865 (43.6%)	
Means of transport — *n* (%)	Ambulance	288 (6.6%)	434 (9.7%)	Ambulance vs independently/other 1.52 (1.31–1.78); < 0.001
	Independently/other	4085 (93.3%)	4039 (90.2%)	
COVID-19 test — *n* (%)	Negative	1 (0.0%)	392 (8.8%)	//
	Positive	0 (0.0%)	0 (0.0%)	
COVID-19 quarantine — *n* (%)	No	4380 (100.0%)	4428 (98.8%)	< 0.001
	Yes	0 (0.0%)	52 (1.2%)	
COVID-19 fiduciary isolation — *n* (%)	No	4380 (100.0%)	4478 (100.0%)	//
	Yes	0 (0.0%)	2 (0.0%)	
Parent/caregiver present during the accident — *n* (%)	No	251 (9.8%)	275 (11.7%)	0.81 (0.68–0.97); 0.025
	Yes	2323 (90.2%)	2068 (88.3%)	
Priority code — *n* (%)	Not specified	117 (2.7%)	43 (1.0%)	//
	White	202 (4.6%)	163 (3.6%)	
	Green	3507 (80.1%)	3802 (84.9%)	
	Yellow	542 (12.4%)	451 (10.1%)	
	Red	12 (0.3%)	21 (0.5%)	
Priority code — *n* (%)	White/green	3709 (87.0%)	3965 (89.4%)	White/green vs yellow/red 1.26 (1.10–1.43); 0.001
	Yellow/red	554 (13.0%)	472 (10.6%)	
Place — *n* (%)	Bathroom	122 (2.8%)	96 (2.1%)	//
	Living room/lounge	25 (0.6%)	42 (0.9%)	
	Garage/other	39 (0.9%)	50 (1.1%)	
	Balcony/garden/terrace	229 (5.2%)	567 (12.7%)	
	Living room/lounge	173 (3.9%)	229 (5.1%)	
	Storage/attic/cellar	0 (0.0%)	5 (0.1%)	
	Stairs (internal or external)	192 (4.4%)	137 (3.1%)	
	Kitchen	152 (3.5%)	160 (3.6%)	
	Hallway/entrance	18 (0.4%)	38 (0.8%)	
	Bedroom/children’s room	364 (8.3%)	416 (9.3%)	
	Unknown/not reported	3066 (70.0%)	2740 (61.2%)	
Place — *n* (%)	Bedroom/children room	364 (27.7%)	416 (23.9%)	Bathroom vs bedroom/children room: 0.69 (0.51–0.93); 0.015 Living room/loung vs bedroom/children room: 1.20 (0.95–1.51); 0.13 Kitchen vs bedroom/children room: 0.92 (0.71–1.20); 0.54 Other vs bedroom/children room: 1.46 (1.22–1.75); < 0.001
	Bathroom	122 (9.3%)	96 (ki5.5%)	
	Living room/lounge	198 (15.1%)	271 (15.6%)	
	Kitchen	152 (11.6%)	160 (9.2%)	
	Other	478 (36.4%)	797 (45.8%)	

Most of patients were males (56% in both periods), with a mean age of 7.1 years (SD ± 3.18) in 2019 and 6.9 years (SD ± 3.02) in 2020.

Patients with a high priority code (yellow/red) for ED visits were significantly reduced from 13% in 2019 to 10% in 2020 (*p* = 0.001), but the number of patients arriving to the ED with the ambulance increased (from 6.6% in 2019 to 9.7% in 2020; *p* < 0.001).

Other demographics, clinical characteristics, and hospital access info of the sample are presented in Table 1.

No child enrolled in 2020 tested positive for SARS-CoV-2.

**Fig. 1:**
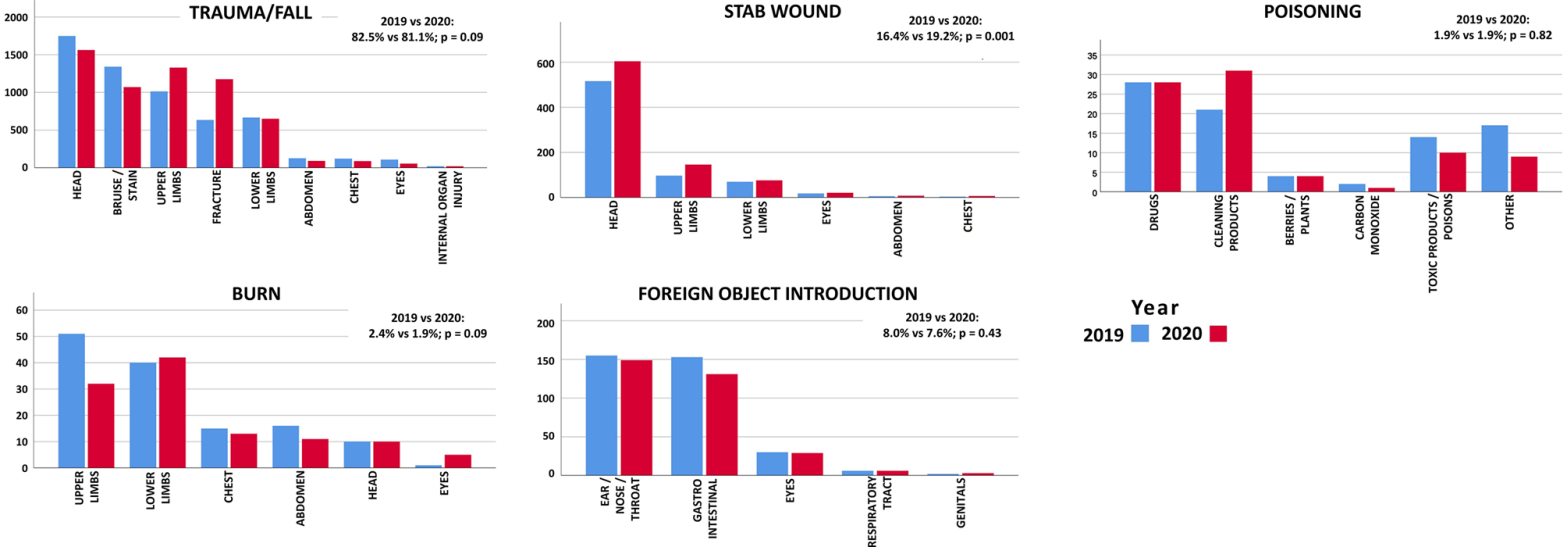
Distribution of different types of accidents during the two observation periods

Considering the mechanism of injury, only stab wound registered a significant change in frequency in 2020, with an increment from 16.4% in 2019 to 19.2% in 2020 (*p* = 0.001). In general, trauma/falls were the most frequent, reported in 82.5% of cases in 2019 and 81.1% in 2020, followed by stab wound and foreign object introduction (8.0% in 2019 and 7.6% in 2020) (Fig. 1).

**Fig. 2:**
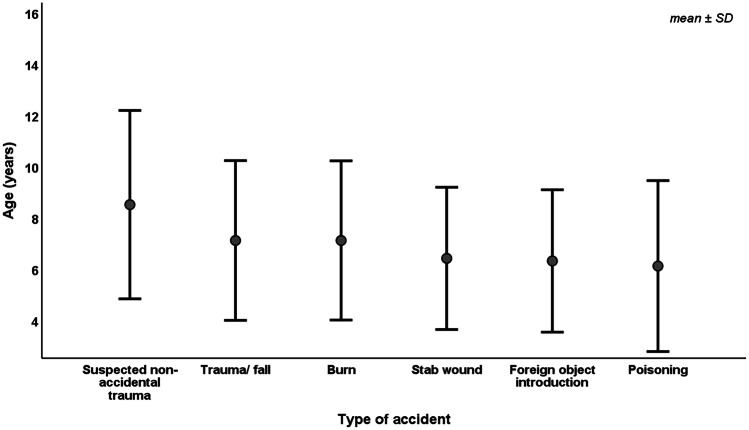
Age of patients by type of accident

We assessed the age difference based on the presence/absence of occurrence of a certain mechanism of injury, and we observed an older age of patients having a suspected non-accidental trauma (presence vs absent 8.5 ± 3.68 vs 7.0 ± 3.10, *p* < 0.001) or a trauma/fall (presence vs absent 7.1 ± 3.12 vs 6.6 ± 3.02, *p* < 0.001). On the contrary, younger patients have received an ED visit for stab wound (presence vs absent 6.4 ± 2.78 vs 7.1 ± 3.16, *p* < 0.001), for a foreign object introduction (presence vs absent 6.3 ± 2.78 vs 7.0 ± 3.12, *p* < 0.001) and for poisoning (presence vs absent: 6.1 ± 3.34 vs 7.0 ± 3.10, *p* < 0.001). No difference was observed for burn accidents (*p* = 0.53) (Appendix — Table 5 and Fig. 2).

**Table 2:** Outcomes

		**2019**	**2020**	***p***
Resuscitation activity — *n* (%)	No	4374 (99.9%)	4474 (99.9%)	0.98 (0.32–3.03); 0.99
	Yes	6 (0.1%)	6 (0.1%)	
If performed. where — *n*	Hospital	2	3	
	Home	3	2	
Outcome — *n* (%)	Discharge	3956 (90.4%)	3914 (87.4%)	Short-stay observation vs discharge: 0.95 (0.77–1.17); 0.61 Hospitalization vs discharge: 1.85 (1.55–2.20); < 0.001 Refusal to be hospitalized vs discharge: 0.26 (0.11–0.65); 0.004
	Short-stay observation	193 (4.4%)	181 (4.0%)	
	Hospitalization	205 (4.7%)	375 (8.4%)	
	Refusal to be hospitalized	23 (0.5%)	6 (0.1%)	
Hospital stays (days) — mean ± SD (range)		1.1 ± 1.44 (1–68)	1.1 ± 1.16 (1–59)	0.98 (0.94–1.01); 0.22
Estimated recovery period (days) — median (range)		5 (0–60)	7 (0–60)	1.04 (1.03–1.04); < 0.001

Patients in 2020 required more frequently diagnostic tests, especially limbs X-ray (from 30.9% in 2019 to 38.5% in 2020, *p* < 0.001), blood sampling (from 4.6% in 2019 to 5.6% in 2020, *p* = 0.026), and surgical therapy (from 13.2% in 2019 to 20.2% in 2020, *p* < 0.001). In the same way, the visits requiring a specialist consultation in ED increased (from 48.2% in 2019 to 57.1% in 2020, *p* < 0.001), whereas prescriptions of medical therapy decreased (from 64.1% in 2019 to 61.6% in 2020, *p* = 0.012) (Appendix — Table 6).

**Table 3:** Factors associated with hospitalization

	**Univariate**	**Multivariate**
Year (2020 vs 2019)	1.86 (1.56–2.22); < 0.001	1.86 (1.55–2.24); < 0.001
Age	1.05 (1.03–1.08); < 0.001	1.07 (1.04–1.10); < 0.001
Sex (males vs females)	1.38 (1.16–1.64); < 0.001	1.37 (1.14–1.65); 0.001
Citizen (foreign vs Italian)	1.19 (0.89–1.58); 0.24	–
Means of transport: ambulance vs independently/other	6.72 (5.52–8.18); < 0.001	6.49 (5.29–7.96); < 0.001
Suspected non-accidental trauma	2.05 (1.02–4.15); 0.045	1.09 (0.49–2.42); 0.83
Trauma/fall vs other reasons	0.65 (0.53–0.79); < 0.001	0.95 (0.62–1.46); 0.82
Stab wound vs other reasons	0.53 (0.41–0.70); < 0.001	0.51 (0.37–0.71); < 0.001
Burn vs other reasons	1.84 (1.16–2.91); 0.010	1.54 (0.82–2.90); 0.18
Foreign object introduction vs other reasons	1.37 (1.04–1.82); 0.027	1.36 (0.82–2.25); 0.24
Poisoning vs other reasons	4.43 (3.05–6.42); < 0.001	3.52 (1.96–6.31); < 0.001

When evaluating the outcomes, we observed a sharp increase in the number of hospitalization (from 4.7% in 2019 to 8.4% in 2020, *p* < 0.001) and in the estimated recovery period (from 5 days in 2019 to 7 days in 2020, *p* < 0.001) (Table 2).

The risk of ED admission with a severe priority code (yellow/red) was higher when the accident occurred without the presence of a parent or caregiver (*OR*: 1.87, 95% CI: 1.48–2.36; *p* < 0.001).

Table 3 reports factors associated to hospitalization. Higher risk of hospitalization was showed in patients accessing during 2020 vs 2019 (*OR*: 1.86, 95% CI: 1.55–2.24; *p* < 0.001), in older patients (*OR*: 1.07, 95% CI: 1.04–1.10; *p* < 0.001) and in males compared to females (*OR*: 1.37; 95% CI: 1.14–1.65; *p* = 0.001). Patients arriving to the ED with the ambulance had a higher risk of hospitalization compared to patients who reached the hospital independently (*OR*: 6.49, 95% CI: 5.29–7.96; *p* < 0.001). Considering the type of injury, we observed a significant higher risk of hospitalization for poisoning (vs other reasons, *OR*: 3.52; 95% CI: 1.96–6.31; *p* < 0.001) and lower risk in case of stab wound (vs other reasons, *OR*: 0.51; 95% CI: 0.37–0.71; *p* < 0.001). In univariate analysis, a relevant effect of suspected non-accidental trauma on hospitalization was found (*OR* 2.05, 95% CI: 1.02–4.15; *p* = 0.045), however, not confirmed in the subsequent multivariate analysis.

## Discussion

To the best of our knowledge, this is the first Italian multicentre study that aims to understand the impact of SARS-CoV-2 lockdown to the ED visits for DA in children.

Despite the sharp decrease in the number of ED visits for all causes observed in children during the lockdown period, confirming other Italian literature data [7–21], the number of visits for DAs remained stable, highlighting higher odds of paediatric visits for DA in 2020 than 2019. This increase of risk of DAs during lockdown period confirms data observed in the previous monocentric/regional studies conducted in Italy [15–17, 19, 20, 22, 23]. It might be explained not only with the growing number of hours spent at home but probably also with the negative psychological effects of COVID-19 home confinement both in children and in parents/caregivers [23]. If lockdown on the one hand concurred to the reduction of total ED visits for the decrease of infectious diseases in children, on the other, it contributed to the deterioration of psychological wellbeing due to home confinement, the lack of personal space, physical activity, and social interaction, not only for children but also in parents. All these negative effects could bring to a reduction of child supervision with an increase of accidents [23].

No difference in the frequency of suspected non-accidental trauma was found between two observation periods; in any case, this kind of data could be underestimated because cases collected in ED databases do not allow to correctly estimate whether children were actual victims of abuse [24].

Regarding the severity of injuries, during 2020, we observed a reduction in the white code visits compared to 2019, indicating an avoidance of unnecessary visits, probably due to the fear about contracting the SARS-CoV-2 in hospitals. During 2020, the number of visits with green code increased compared to 2019, probably due to COVID-19 prevention measures with reduction of outpatient activities in most of paediatric hospitals.

A recent study has shown that the increase of hospitalization might be explained by change of hospital protocol for injury treatment at ED. If before SARS-CoV-2 pandemic many operative procedures could be performed directly at the ED, during lockdown, patients were hospitalized to receive the same procedures [23]. However, this change in patient management was not confirmed by all centres participating in this study.

Regarding factors increasing odds of being hospitalized, poisoning represented the major risk, although it is responsible for a very small part of all ED admissions, and principal causes were drugs and cleaning products. In fact, in younger children, the ingestion of drugs could be due to the imitation of adult behaviours, while for cleaning products, particular attention should be paid to colourful ones and easy-to-open containers [25].

## Conclusions

The present study has a main limitation which should be mentioned. Data were collected retrospectively and were only available for patients who presented at the hospital. This finding shows possible differences in ED accesses between the two observation periods, but not in the total number of incidents that truly occurred. Certainly, there may be an underestimation in the number of milder incidents, which, during the lockdown, may have been handled at home and not in hospital. In addition, we have to consider that this is a retrospective study and, as such, we could not retrieve missing data. The analysis of factors associated with hospitalization was limited to variables collected in the medical records. It would be interesting to investigate the cases of suspected non-accidental incidents, an analysis that we will reserve for a new project.

In any case, our study confirms, at national level, that SARS-CoV-2 lockdown had a high impact on paediatric ED visits for DA, and on hospitalization too. In fact, although the home confinement was a successful strategy to prevent the diffusion of SARS-CoV-2, it seems to have a negative impact on the risk of DA.

Regarding risk of injuries at home and, in particular, poisoning, a public health campaign aimed at caregivers would be necessary to minimize possible risks at home.

## Data Availability

The data that support the findings of this study are available from the corresponding author, DB, upon reasonable request.

## Appendix

**Table 4:** Incidence rate of domestic injuries out of total number of accesses

	**Incidence rate (95%CI)**		**IRR (95%CI)**	***p***
	**2019**	**2020**		
Azienda Ospedaliera Universitaria Integrata Verona	0.036 (0.031–0.044)	0.093 (0.077–0.111)	2.531 (1.963–3.262)	< 0.001
IRCCS Burlo Garofolo di Trieste	0.075 (0.068–0.083)	0.234 (0.212–0.257)	3.118 (2.708–3.591)	< 0.001
A.O.U. Città della Salute e della Scienza di Torino	0.033 (0.030–0.037)	0.097 (0.088–0.107)	2.935 (2.533–3.402)	< 0.001
ASL 2 Savonese – Ospedale San Paolo—Savona	0.037 (0.031–0.044)	0.109 (0.085–0.140)	2.961 (2.154–4.039)	< 0.001
IRCCS Ospedale Pediatrico Bambino Gesù—Roma	0.064 (0.059–0.070)	0.267 (0.251–0.284)	4.167 (3.753–4.630)	< 0.001
Ospedale San Carlo – Potenza	0.029 (0.024–0.035)	0.069 (0.053–0.088)	2.374 (1.716–3.266)	< 0.001
Meyer—Azienda Ospedaliero Universitaria—Firenze	0.056 (0.051–0.060)	0.257 (0.241–0.274)	4.631 (4.177–5.138)	< 0.001
AO Brotzu – Ospedale San Michele—Cagliari	0.170 (0.158–0.183)	0.305 (0.275–0.337)	1.791 (1.579–2.030)	< 0.001
Ospedale Sant'Orsola di Bologna	0.048 (0.042–0.055)	0.081 (0.068–0.096)	1.673 (1.349–2.070)	< 0.001
AUSL Bologna – Ospedale Maggiore	0.030 (0.025–0.035)	0.124 (0.107–0.144)	4.194 (3.370–5.225)	< 0.001
Ospedale Papa Giovanni XXIII—Bergamo	0.028 (0.024–0.034)	0.118 (0.101–0.136)	4.119 (3.286–5.172)	< 0.001
Clinica Pediatrica Udine – ASUFC*	0.083 (0.075–0.092)	0.198 (0.175–0.222)	2.144 (1.830–2.509)	< 0.001
**Total cases**	0.056 (0.054–0.058)	0.179 (0.173–0.184)	3.158 (3.022–3.299)	< 0.001

**Table 5:** Age difference (years) based on the presence/absence of occurrence of a certain accident

	**Present**	**Absent**	***p***
Suspected non-accidental trauma — mean ± SD	8.5 ± 3.68	7.0 ± 3.10	< 0.001
Trauma/fall — mean ± SD	7.1 ± 3.12	6.6 ± 3.02	< 0.001
Stab wound — mean ± SD	6.4 ± 2.78	7.1 ± 3.16	< 0.001
Foreign object introduction — mean ± SD	6.3 ± 2.78	7.0 ± 3.12	< 0.001
Poisoning — mean ± SD	6.1 ± 3.34	7.0 ± 3.10	< 0.001
Burn — mean ± SD	7.1 ± 3.11	7.0 ± 3.10	0.53

**Table 6:** Diagnostic tests, therapies, and specialist advice

		**2019**	**2020**	***p***
Ultrasound — *n* (%)	No	4260 (97.3%)	4326 (96.6%)	0.06
	Yes	120 (2.7%)	154 (3.4%)	
CT scan — *n* (%)	No	4287 (97.9%)	4368 (97.5%)	0.24
	Yes	93 (2.1%)	112 (2.5%)	
Chest X-ray — *n* (%)	No	4234 (96.7%)	4344 (97.0%)	0.43
	Yes	146 (3.3%)	136 (3.0%)	
Abdomen X-ray — *n* (%)	No	4266 (97.4%)	4370 (97.5%)	0.66
	Yes	114 (2.6%)	110 (2.5%)	
Limbs X-ray — *n* (%)	No	3027 (69.1%)	2754 (61.5%)	< 0.001
	Yes	1353 (30.9%)	1726 (38.5%)	
ECG — *n* (%)	No	4317 (98.6%)	4414 (98.5%)	0.89
	Yes	63 (1.4%)	66 (1.5%)	
Blood sampling — *n* (%)	No	4180 (95.4%)	4229 (94.4%)	0.026
	Yes	200 (4.6%)	251 (5.6%)	
Other diagnostic procedure — *n* (%)	No	4059 (92.7%)	4201 (93.8%)	0.035
	Yes	321 (7.3%)	278 (6.2%)	
Surgical therapy required — *n* (%)	No	3798 (86.8%)	3574 (79.8%)	< 0.001
	Yes	580 (13.2%)	906 (20.2%)	
Prescribed medical therapy — *n* (%)	No	1570 (35.9%)	1721 (38.4%)	0.012
	Yes	2809 (64.1%)	2758 (61.6%)	
Specialist consultation required — *n* (%)	No	2268 (51.8%)	1920 (42.9%)	< 0.001
	Yes	2111 (48.2%)	2558 (57.1%)	

## Abbreviations

DA: Domestic accident
ED: Emergency department
IRR: Incidence rate ratio

## References

[1] da Silva MF, da Fontinele DR, de Oliveira AVS (2017). Determining factors of domestic accidents in early childhood. Journal of Human Growth and Development.

[2] EpiCentro Oms e Unicef: la prevenzione degli infortuni e degli incidenti nei bambini. https://www.epicentro.iss.it/incidenti/infanziaOms08. Accessed 17 Jan 2023

[3] La sorveglianza Siniaca. In: ISS. https://iss.it/siniaca-la-sorveglianza-siniaca. Accessed 17 Jan 2023

[4] EpiCentro Sicurezza domestica dati sorveglianza Passi. https://www.epicentro.iss.it/passi/dati/SicurezzaDomestica. Accessed 17 Jan 2023

[5] Kruizinga MD, Peeters D, van Veen M et al (2021) The impact of lockdown on pediatric ED visits and hospital admissions during the COVID19 pandemic: a multicenter analysis and review of the literature. Eur J Pediatr 180:2271–2279. 10.1007/s00431-021-04015-0 10.1007/s00431-021-04015-0 PMC7959585 33723971

[6] Tsoumakas K, Dousis E, Mavridi F (2009). Parent’s adherence to children’s home-accident preventive measures. Int Nurs Rev.

[7] Cella A, Marchetti F, Iughetti L et al (2020) Italian COVID-19 epidemic: effects on paediatric emergency attendance-a survey in the Emilia Romagna region. BMJ Paediatr Open 4:e000742. 10.1136/bmjpo-2020-000742 10.1136/bmjpo-2020-000742 PMC10577789 34192169

[8] Ciacchini B, Tonioli F, Marciano C (2020). Reluctance to seek pediatric care during the COVID-19 pandemic and the risks of delayed diagnosis. Ital J Pediatr.

[9] Cozzi G, Zanchi C, Giangreco M (2020). The impact of the COVID-19 lockdown in Italy on a paediatric emergency setting. Acta Paediatr.

[10] Lazzerini M, Barbi E, Apicella A (2020). Delayed access or provision of care in Italy resulting from fear of COVID-19. Lancet Child Adolesc Health.

[11] Matera L, Nenna R, Rizzo V (2020). SARS-CoV-2 pandemic impact on pediatric emergency rooms: a multicenter study. Int J Environ Res Public Health.

[12] Valitutti F, Zenzeri L, Mauro A (2020). Effect of population lockdown on pediatric emergency room demands in the era of COVID-19. Front Pediatr.

[13] Vierucci F, Bacci C, Mucaria C (2020). How COVID-19 pandemic changed children and adolescents use of the emergency department: the experience of a secondary care pediatric unit in Central Italy. SN Compr Clin Med.

[14] Morello F, Bima P, Ferreri E (2021). After the first wave and beyond lockdown: long-lasting changes in emergency department visit number, characteristics, diagnoses, and hospital admissions. Intern Emerg Med.

[15] Iozzi L, Brambilla I, Foiadelli T (2020). Paediatric emergency department visits fell by more than 70% during the COVID-19 lockdown in Northern Italy. Acta Paediatr.

[16] Mataloni F, Colais P, Pinnarelli L et al (2022) The impact of the SARS COV-2 pandemic on pediatric accesses in ED: a healthcare emergency information system analysis. PLoS One 17:e0272569. 10.1371/journal.pone.0272569 10.1371/journal.pone.0272569 PMC9355200 35930569

[17] Raffaldi I, Castagno E, Fumi I et al (2021) Pediatric admissions to emergency departments of North-Western Italy during COVID-19 pandemic: a retrospective observational study. Lancet Reg Health Eur 5:100081. 10.1016/j.lanepe.2021.100081 10.1016/j.lanepe.2021.100081 PMC7969147 34104902

[18] Clavenna A, Nardelli S, Sala D (2020). Impact of COVID-19 on the pattern of access to a pediatric emergency department in the Lombardy Region, Italy. Pediatr Emerg Care.

[19] Liguoro I, Pilotto C, Vergine M (2021). The impact of COVID-19 on a tertiary care pediatric emergency department. Eur J Pediatr.

[20] Raucci U, Musolino AM, Di Lallo D (2021). Impact of the COVID-19 pandemic on the emergency department of a tertiary children’s hospital. Ital J Pediatr.

[21] Masetti R, Corsini I, Leardini D et al (2020) Presentations to the emergency department in Bologna, Italy, during COVID-19 outbreak. BMJ Paediatr Open 4:e000748. 10.1136/bmjpo-2020-000748 10.1136/bmjpo-2020-000748 PMC7372170 34192170

[22] Bressan S, Gallo E, Tirelli F et al (2021) Lockdown: more domestic accidents than COVID-19 in children. Arch Dis Child 106:e3. 10.1136/archdischild-2020-319547 10.1136/archdischild-2020-319547 32487724

[23] Ferro V, Nacca R, Pisani M (2022). Children at risk of domestic accidents when are locked up at home: the other side of COVID-19 outbreak lockdown. Ital J Pediatr.

[24] Castagnino M, Paglino A, Berardi C et al (2020) Recording risk factors of physical abuse in children younger than 36 months with bone fractures: a 12-years retrospective study in an Italian general hospital emergency room. Front Ped 8:183. 10.3389/fped.2020.00183 10.3389/fped.2020.00183 PMC7186300 32373567

[25] Soave PM, Curatola A, Ferretti S et al (2022) Acute poisoning in children admitted to pediatric emergency department: a five-years retrospective analysis. Acta Biomed 93:e2022004. 10.23750/abm.v93i1.11602 10.23750/abm.v93i1.11602 PMC8972869 35315415

