# Correction to: Frequency and type of domestic injuries among children during COVID-19 lockdown: what changes from the past? An Italian multicentre cohort study

**DOI:** 10.1007/s00431-023-05060-7

**Published:** 2023-07-08

**Authors:** Daiana Bezzini, Marcello Lanari, Alessandro Amaddeo, Melodie O. Aricò, Emanuele Castagno, Gabriella Cherchi, Giulia Giacomini, Giulia Graziani, Salvatore Grosso, Ilaria Liguoro, Francesca Lombardi, Sergio Manieri, Laura Moschettini, Francesca Parisi, Antonino Reale, Giulia Romanisio, Davide Silvagni, Irene Schiavetti, “Keep Me Safe” study group, Alberto Gaiero, Alessandra Iacono, Alessandro Amaddeo, Alessandro Canetto, Alice Fachin, Angela Demarco, Annalisa Lo Sasso, Annalisa Rossetti, Antonino Reale, Arianna Dagri, Carmela G. Raffaele, Chiara Ghizzi, Claudia Bondonez, Daiana Bezzini, Daniele Zama, Davide Silvagni, Elisa Pala, Elisabeth Pangallo, Emanuele Castagno, Enrico Valerio, Enrico Valleta, Federico Marchetti, Francesca Lombardi, Francesca Nicolardi, Francesca Parisi, Francesco Medici, Francesco Silenzi, Gabriella Cherchi, Giulia Ceccarini, Giulia Giacomini, Giulia Graziani, Giulia Romanisio, Ilaria Corsini, Ilaria Liguoro, Irene Frigo, Irene Raffaldi, Irene Schiavetti, Laura Andreozzi, Laura Moschettini, Laura Penta, Luca Bianchini, Luciana Romaniello, Manuel Murciano, Manuela Pagano, Marcello Lanari, Maria Chiara Supino, Maria Pia Mirauda, Martina Scilipoti, Matteo Calvi, Melodie O. Arico, Monia Gennari, Nicoletta Della Vecchia, Paolo Biban, Paolo Tarlazzi, Raffaele Pecoraro, Raffaella Nacca, Rosa Francavilla, Rosa Lapolla, Salvatore Grosso, Sergio Manieri, Silvia Carlassara, Simone Ajello, Stefano Masi, Viola Carzaniga

**Affiliations:** 1 Department of Life Sciences, University of Siena, via Aldo Moro 2, 53100 Siena, Italy; 2 Pediatric Emergency Unit, IRCCS Azienda Ospedaliera Universitaria di Bologna, Bologna, Italy; 3 Institute for Maternal and Child Health, IRCCS “Burlo Garofolo”, Trieste, Italy; 4 Pediatric Department, G.B. Morgagni - L. Pierantoni Hospital, Forlì, Italy; 5 Department of Paediatric Emergency, Regina Margherita Children’s Hospital - A.O.U. Città della Salute e della Scienza di Torino, Turin, Italy; 6 Ospedale San Michele, ARNAS “G.Brotzu”, Cagliari, Italy; 7 IRCCS Meyer University Children’s Hospital, Florence, Italy; 8 Department of Paediatrics, Santa Maria delle Croci Hospital, Ravenna, Italy; 9 Clinical Paediatrics, Department of Molecular Medicine and Development, University of Siena, Siena, Italy; 10 Division of Paediatrics, Department of Medicine (DAME), University of Udine, Udine, Italy; 11 Pediatric Clinic, “Santa Maria della Misericordia” University Hospital - Azienda Sanitaria Universitaria Friuli Centrale, Udine, Italy; 12 Pediatric Department, Maggiore Hospital Carlo Alberto Pizzardi, Bologna AUSL, Bologna, Italy; 13 Department of Paediatrics, San Carlo Hospital, Potenza, Italy; 14 Pediatric Department, University of Milano Bicocca, Milan, Italy; 15 Department of Medicine and Surgery, Pediatric Clinic, University of Perugia, Perugia, Italy; 16 Department of Emergency and General Paediatrics, Bambino Gesù Children’s Hospital, IRCCS, Rome, Italy; 17 Pediatric and Neonatology Unit, ASL 2 Ospedale San Paolo, Savona, Italy; 18 Department of Paediatric Emergency, Azienda Ospedaliera Universitaria Integrata Verona, Verona, Italy; 19 Department of Health Sciences, University of Genoa, Genoa, Italy

**Correction to: European Journal of Pediatrics** **https://doi.org/10.1007/s00431-023-04990-6**

The authors under the "Keep Me Safe" study group in the original published version of the above article should have been presented in the authorship section.

The original article has been corrected.

